# Supplementary material for: NetMedPy: a Python package for large-scale network medicine screening
Source: Bioinformatics. 2025 Jun 17;41(9):btaf338. doi: 10.1093/bioinformatics/btaf338 (PMC12401583; doi:10.1093/bioinformatics/btaf338)
Supplement: btaf338_Supplementary_Data [file btaf338_supplementary_data.pdf]

NetMedPy: Supplementary Information

Table of Contents

SI. Network Medicine Background **2**

SI.I. Disease Modules 2

SI.II. Proximity 2

SI.III. Separation 3

SII Null Models in NetMedPy **4**

SIII. Information passing metrics **5**

SIII.I Random Walks with Restart 6

SIII.II Biased Random Walks with Restart 7

SIII.III Communicability 7

SIII.IV Converting association scores into distances 8

SIV. Data Acquisition **8**

SIV.I Protein-Protein Interaction Networks 8

SIV.II Selection of Vitamin D Targets 9

SIV.III Selection of Gene-Disease Associations 10

SV. NetMedPy Installation **10**

SVI. Robustness Study with NetMedPy **12**

SVI. Supplementary Figures **17**

Agreement between metrics for the AMSPL 17

SI. Network Medicine Background

SI.I Disease Modules

Different studies have shown that genes associated with a particular disease tend to cluster within the same network neighborhood, frequently forming a connected sub-network within the interactome[1,13,15]. The largest connected component (LCC) formed by these disease genes contains important information about the disease localization within the interactome, as well as the main interactions between the disease genes[15]. Studying the LCC allows researchers to efficiently narrow down the vast number of potential biomarkers to those most likely to be clinically relevant in drug discovery and repurposing efforts.

Calculating the statistical significance of the LCC size in Network Medicine is the standard framework for assessing the relevance of disease gene localization. For any set of nodes in the interactome, the statistical significance of the LCC size is determined by the Z-Score of the observed size l:

$$Z = \frac{l - \mu_l}{\sigma_l}, \tag{1}$$

where μ_l is the expected LCC size for nodes chosen randomly, and σ_l is the LCC standard deviation across the randomized ensemble. Additionally, the p-value associated to l is calculated as the probability of observing an LCC of size l or superior in a random sample of nodes. These two indicators are important in their ability to validate the biological relevance of the LCC, ensuring that the connections and interactions within the module are not random artifacts but likely reflect accurate biological processes and pathways implicated in the disease.

SI.II Proximity

Given X, a set of disease genes and Y, a set of drug targets, we define $d_c(X, Y)$ as the average shortest path length between the drug's targets and the nearest disease node[6]:

$$d_c(X, Y) = \frac{1}{|Y|} \sum_{y \in Y} \min_{x \in X} d(x, y), \tag{2}$$

where $d(x, y)$ is the length of the shortest path between node x and y. For each drug-disease pair, proximity assesses whether the drug targets are closer to the disease nodes than expected if both sets were randomly chosen across the network. The statistical significance of the distance d_c is determined by calculating the Z-score of the observed drug-disease distance compared with the respective random expectation:

$$Z = \frac{d_c - \mu}{\sigma}. \tag{3}$$

Large negative scores indicate that the drug targets are closer to disease nodes than random, while large positive scores indicate the drug targets are farther away from disease nodes than expected.

The p-value of d_c is calculated as the probability of observing a distance of length d_c or inferior in random samples of drug targets and disease nodes. Note that Eq. (2) is not symmetric, since $d_c(X, Y) \neq d_c(Y, X)$.

To broaden the applicability of proximity beyond its original use in drug discovery, we propose a straightforward symmetric proximity measure by replacing d_c in Eq. (2) with the average minimum shortest path length in both directions:

$$d_s(X, Y) = \frac{d_c(X, Y) + d_c(Y, X)}{2}. \tag{4}$$

Then, μ and σ in Eq. (3) are calculated using a null model that considers the definition of d_s in Eq. (4).

SI.III Separation

Similar to proximity, separation was introduced as a relative measure of distance between node sets within a network, with its primary application being the identification of disease comorbidities[12]. Indeed, if two disease modules overlap, perturbations leading to one disease are most likely to propagate to the other disease module, disrupting pathways and biological processes involved in both diseases and resulting in shared abnormalities and phenotypes. The separation between disease A and disease B is defined as

$$S_{AB} = d_{AB} - \frac{d_{AA} + d_{BB}}{2}, \tag{5}$$

where d_{AB} represents the average of the shortest path length between all genes of disease A and all genes of disease B. Hence, S_{AB} compares the shortest distance between genes of diseases A and B to the within-disease-module distance d_{AA} and d_{BB}. Negative separation values indicate that the average shortest path distance between the modules is smaller than the "average size" of the two modules, suggesting the presence of shared mechanisms or risk factors.

SII. Null Models in NetMedPy

NetMedPy offers the following null models to estimate the significance of the LCC, proximity, and separation:

- **Perfect Degree Match.** This model selects a random sample replicating the original node-set's degree distribution. A node's degree, indicating its number of connections, is a highly predictive network property. By preserving this distribution in the sample, the null model ensures that the random set is structurally similar to the original, allowing to search for patterns not solely driven by the degree.

- **Logarithmic Binning.** This model involves categorizing the degrees of all nodes

within the network into logarithmically sized bins, with each bin containing a fixed number of nodes. Samples are then drawn by matching the degree of the original nodes to those within the corresponding bins. This method allows more variability for high degree nodes, providing a more generalized yet representative sample for comparison.

- **Strength Binning.** Analogous to logarithmic binning, strength binning uses the strength of the nodes—typically a weighted sum of their connections—instead of their degrees. This model is particularly useful in weighted networks where the strength of the nodes might provide a more accurate reflection of their functional significance.

- **Uniform Distribution.** This model randomly selects nodes from the entire network, disregarding their degree or strength. It represents an entirely random sampling approach, providing a baseline for evaluating the extent to which the original node set's structural or functional features influence the observed metrics.

- **Custom Null Model.** This feature allows users to specify their null model, enabling the application of unique or novel statistical frameworks adapted to specific research questions or datasets. It provides methodological flexibility and can be particularly valuable in cases where standard null models do not adequately address the particularities of the data.

SIII. Information passing metrics

Proximity and separation rely on the definition of a "distance" between nodes, measured as the length of their shortest path, to quantify how information traverses the interaction network.

Beyond the shortest path, NetMedPy includes three additional methodologies to quantify information flow within the network: random walks with restart (RWR), biased random walks with restart (BRWR), and communicability. Each method calculates an association

score $\phi(a, b)$, reflecting how effectively information can be transmitted between node a and b. Large $\phi(a, b)$ scores in any of these measures suggest a strong potential for interaction or functional relationship between nodes. Consequently, we redefined the distance between nodes based on $\phi(a, b)$, ensuring that higher scores correspond to shorter distances within the network.

SIII.I Random Walks with Restart

Random Walks with Restart (RWR) simulate the random movement of molecules or signals through a network, allowing the walker to return to a starting node with a certain probability at each step. This approach captures the stochastic nature of biological processes while emphasizing the local context of the starting node. The restart mechanism ensures that the walker frequently returns to the initial node, preventing it from drifting too far and providing a more accurate reflection of node importance within the vicinity. This makes RWR valuable for understanding biological networks' local and global properties and identifying key regulatory nodes and potential therapeutic targets[8].

Let A be the adjacency matrix of the network, D be the diagonal degree matrix where $D_{ii} = \sum_j A_{ij}$, P be the transition probability matrix given by $P = AD^{-1}$, and α be the restart probability at node a. The steady-state probability distribution r_a of a random walk restarting at node a is defined as[2,11]:

$$r_a = \alpha(I - (1 - \alpha)P)^{-1}p, \tag{6}$$

where p is the restart distribution vector, typically a unit vector with 1 at the starting node and 0s elsewhere. Specifically, each element r_{ai} of the vector r_a corresponds to the steady-state probability of being at node i after a large number of steps in the random walk process, considering the possibility of restarting at the initial node a with probability α. The association score $\phi_{RWR}(a, b)$ for RWR is simply:

$$\phi_{RWR}(a,b) = r_{ab}. \tag{7}$$

SIII.II Biased Random Walks with Restart

Biased Random Walks with Restart (BRWR) enhance RWR analysis by incorporating node-specific biases to mitigate the influence of highly connected nodes[3]. We achieve this by dividing the steady-state probability r_{ab} in Eq. (7) by the degree of node b, k_b. Then, the association score $\phi_{BRWR}(a,b)$ for BRWR is calculated as:

$$\phi_{BRWR}(a,b) = \frac{r_{ab}}{k_b}. \tag{8}$$

This technique effectively reduces the bias towards highly connected nodes, providing a more balanced and nuanced understanding of node significance and interaction patterns. BRWR is particularly useful for identifying influential nodes that might not be apparent in the standard RWR.

SIII.III Communicability

Communicability considers all possible paths between nodes, weighted by their lengths, thus providing a comprehensive view of their interaction potential. This measure evaluates pathway redundancy by identifying how perturbations can propagate through indirect interactions, offering a deeper understanding of systemic effects in the network[4,5]. The association score $\phi_{COM}(a,b)$, defined as the communicability between nodes a and b, follows as:

$$\phi_{COM}(a,b) = \sum_{l=0}^{\infty} \frac{(A^l)_{ab}}{l!} = (e^A)_{ab}, \tag{9}$$

where l is the length of the path.

SIII.IV Converting association scores into distances

Since large values of the association score $\phi(a,b)$ are indicative of strong or close interaction between nodes, we define the ϕ-score based "distance" $d_\phi(a,b)$ by inverting and normalizing $\phi(a,b)$:

$$d_\phi(a,b) = 1 - \frac{log(\phi(a,b)) - m}{M - m}, \tag{10}$$

where $m = min_{a,b}\{log(\phi(a,b))\}$ and $M = max_{a,b}\{log(\phi(a,b))\}$. In this way, the distance $d_\phi(a,b)$ assigns small values to nodes with strong association score $\phi(a,b)$, and values close to 1 as $\phi(a,b)$ decreases.

SIV. Data Acquisition

SIV.I Protein-Protein Interaction Networks

The main protein-protein interaction (PPI) network was extracted and integrated from[9],[7], and[10]. Broadly, Luck et al.[9] generated a systematic map of human binary PPIs by conducting nine large-scale yeast two-hybrid (Y2H) screens across a near-complete human ORFeome. The resulting network comprises over 52,000 high-confidence, direct interactions among approximately 8,200 proteins. Huttlin et al.[7] employed large-scale affinity purification-mass spectrometry (AP-MS) to build two proteome-scale, cell-type-specific PPI networks, based on over 15,000 pulldowns performed in HEK293T and HCT116 cells. This approach revealed more than 140,000 interactions, capturing both conserved protein modules and extensive cell-specific interactome variation. Finally, Maron et al.[10] constructed individualized PPI networks (termed reticulotypes) by integrating transcriptomic profiles from patient myocardial samples. Gene pairs whose co-expression patterns significantly diverged from healthy controls were mapped onto a consolidated human interactome, enabling the construction of patient-specific networks that reflect personalized pathobiology and clinical heterogeneity in

hypertrophic cardiomyopathy. The integration of proteins and interactions from these three sources resulted in a comprehensive PPI network containing 18,642 proteins and 354,650 interactions, which serves as the foundation for our main analyses.

For the robustness analysis described in Section *SVI, Robustness Study with NetMedPy*, we utilized two additional PPI networks derived from independent sources. The first was obtained from the BioGRID database (`https://thebiogrid.org/`), comprising 15,451 proteins and 273,908 interactions. The second was sourced from the STRING database (`https://string-db.org/`), where interactions were filtered using a combined score threshold greater than 0.3. This filtering resulted in a network consisting of 18,642 proteins and 710,155 interactions. In both cases, we kept only the proteins and interactions contained in the largest connected component of the entire network to ensure connectivity in the interactome. The complete extraction pipeline for both the BioGRID and STRING networks is publicly available in the GitHub repository at: `https://github.com/menicgiulia/NetMedPy/tree/main/examples/VitaminD/supplementary/sup_code/data_integration/BioNets.ipynb`

SIV.II Selection of Vitamin D Targets

Vitamin D's drug targets were extracted using the CPIExtract package, as detailed in[14]. In brief, CPIExtract extracts, filters, and harmonizes raw data from nine databases: BindingDB (BDB), ChEMBL, Comparative Toxicogenomics Database (CTD), DrugBank (DB), DrugCentral (DC), Drug Target Commons (DTC), Open Targets Platform (OTP), PubChem, and STITCH. For Vitamin D, we find a total of 23 targets mapped to the selected version of the human interactome. The full extraction pipeline is available in the project repository at: `https://github.com/menicgiulia/NetMedPy/tree/main/examples/VitaminD/supplementary/sup_code/data_integration/Vit_D_Targets.ipynb`

SIV.III Selection of Gene-Disease Associations

Gene-disease associations (GDAs) for each disease were extracted and standardized from DisGeNet, Phenopedia, and Open Targets. To ensure that only associations with strong experimental support were retained, we applied the filtering criteria specified in Table S1.

GDA Source	Association Score	Value
Phenopedia	Total Publications	1.00
Open Targets	Overall Association Score	0.10
DisGeNet	Score GDA	0.01

Table S1: Filters applied to produce the GDAs from each source

To balance the number of GDAs from each source, we adjusted these thresholds for Inflammation, COPD (Chronic Obstructive Pulmonary Disease) and CAD (Coronary Artery Disease), according to the values described in Table S2.

Disease	Source	Association Score	Value
Inflammation	Phenopedia	Total publications	4.00
COPD	DisGeNet	Score GDA	0.20
CAD	DisGeNet	Score GDA	0.05

Table S2: Filter criteria for specific diseases, to balance the number of disease genes from each source.

The final list of Gene - Disease Associations has been manually curated by experts in the field, producing a high-quality association list.

SV. NetMedPy Installation

NetMedPy can be installed locally either from GitHub or using pip. To install from GitHub, navigate to the repository at https://github.com/menicgiulia/NetMedPy and follow the instructions in the README file. Alternatively, you can install the package using pip with the following command:

```
pip install netmedpy
```

The package is designed to run in a Python environment above 3.6 and below 3.12, ensuring full functionality of all features. For detailed installation guidelines and additional information, refer to the documentation available on the GitHub repository.

SVI. Robustness Study with NetMedPy

To evaluate the robustness of our network-based findings regarding Vitamin D's association with disease genes, we performed a systematic analysis assessing how different Protein-Protein Interaction (PPI) networks and data incompleteness, such as the genes associated to a disease or the targets associated to a drug, affect the proximity between Vitamin D targets and disease-associated genes. This evaluation is essential to validate the reproducibility and reliability of network medicine studies across different network topologies and data sources.

1. Robustness under different PPI networks.

We compared proximity results obtained from three distinct PPI networks:

- **Main PPI**: A manually curated network integrating multiple public databases. The main results reported in this paper are calculated with this network.
- **BIOGRID PPI**: Derived from BIOGRID. No filter was applied, and all interactions were conserved.
- **STRING PPI**: Extracted from STRING, filtered for interactions with a combined score > 0.3.

Although PPI networks share a substantial number of nodes and a significant portion of edges, they exhibit differences in structure (Figure S1). These discrepancies arise from the distinct data sources, curation methods, and confidence thresholds used to construct each network. To ensure that proximity calculations are based on a connected graph, we extracted the Largest Connected Component (LCC) from each PPI network prior to analysis.

For each PPI network, we calculated the proximity between the set of Vitamin D targets and disease-associated genes (Figure S2). The results indicate that the proximity values are consistent across different network structures. To further assess the robustness of disease

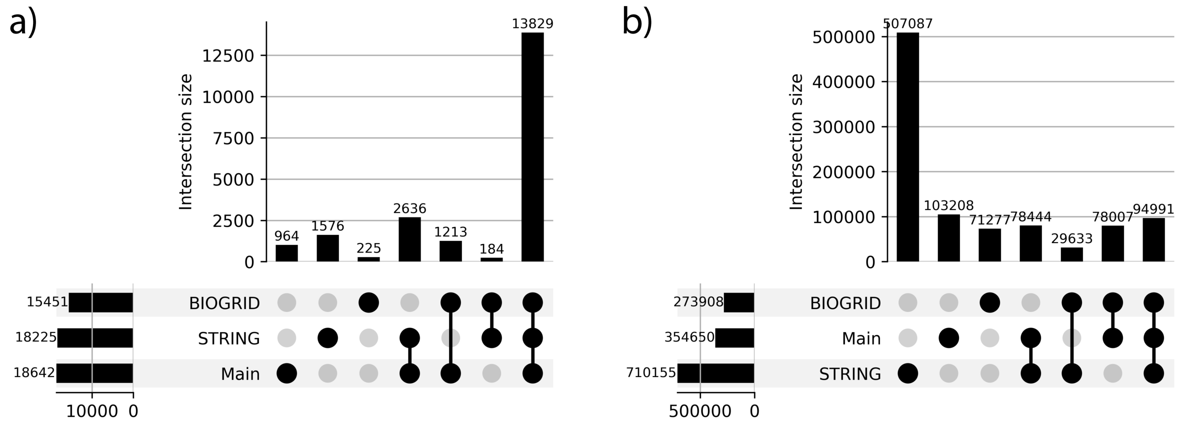

Figure S1: **Node and edge overlap across PPI networks.** The plots illustrate the extent of overlap in protein-coding genes (a) and interactions (b) among the Main, BIOGRID, and STRING PPI networks. While significant overlap is observed, each network also contains unique interactions.

prioritization, we calculated the Spearman Rank Correlation Coefficients (ρ) between proximity rankings derived from each pair of PPI networks (Figure S3). The high correlations observed ($\rho \geq 0.74$) confirm that the relative importance of Vitamin D on diseases remains stable, despite differences in the underlying network data.

Robustness to Target Incompleteness

To further evaluate the stability of Vitamin D - Disease associations, we analyzed how proximity values change when the set of 23 Vitamin D targets is randomly subsampled. This scenario simulates the real-world limitation of incomplete or uncertain knowledge about drug-target interactions.

At each subsampling level $p \in \{20\%, 40\%, 60\%, 80\%\}$, we randomly subsampled $p\%$ of the original Vitamin D targets and recalculated the proximity values to disease-associated genes. This procedure was repeated 10 times per level to ensure statistical reliability.

We calculated the deviation in proximity for each disease i at sampling level p as:

$$\Delta P_i(p) = P_i(p) - P_i(100\%)$$

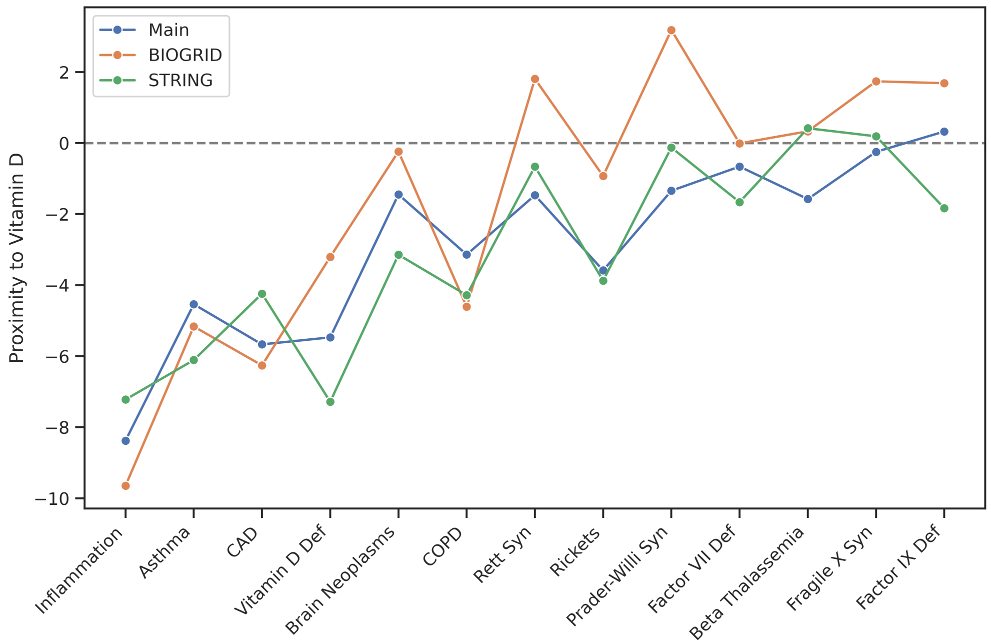

Figure S2: **Proximity to Vitamin D targets across PPI networks.** For each disease, proximity values between Vitamin D targets and disease genes are calculated on the three PPI networks. The consistency of proximity values supports the robustness of the results.

where $P_i(p)$ is the average proximity between the subsampled Vitamin D targets and disease i; $P_i(100\%)$ is proximity calculated using the complete set of Vitamin D targets; and $\Delta P_i(p)$ quantifies the deviation introduced by target incompleteness.

Small deviations ($\Delta P_i(p) \approx 0$) across diseases indicate that proximity values are robust to missing target genes, whereas large deviations suggest sensitivity to the completeness of drug-target information. Our results show that $\Delta P_i(p)$ increases as the proportion of retained Vitamin D targets decreases, corroborating that the inferred relationship between Vitamin D and disease i weakens with fewer known targets (Figure S4). This effect is particularly noticeable for diseases with a strong baseline proximity to Vitamin D, such as Inflammation, Coronary Artery Disease (CAD), and Vitamin D Deficiency. However, with the exception of Inflammation, reducing the target set by up to 20% (i.e., retaining 80% of targets) increases proximity values by less than one unit. This suggests that the strength of the association between Vitamin D and most diseases is robust to moderate levels of target incompleteness.

While absolute proximity values may vary with changes in the input data, it is essential

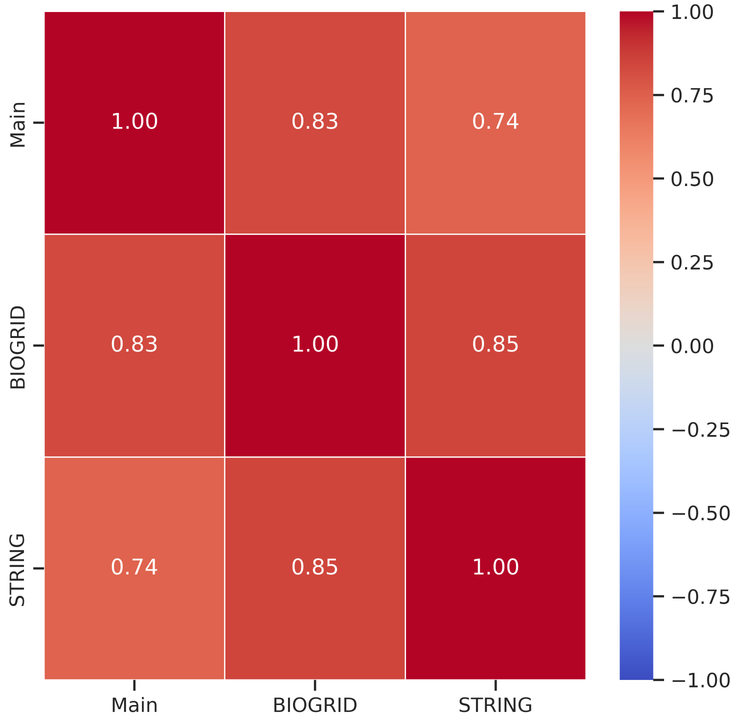

Figure S3: **Spearman rank correlations of proximity scores across PPI networks.** Each pairwise comparison (Main–BIOGRID, Main–STRING, BIOGRID–STRING) shows a strong Spearman correlation ($\rho \geq 0.74$), indicating robust ranking of Vitamin D relevance to diseases, regardless of network choice.

to evaluate whether the relative relevance of Vitamin D across diseases remains stable. To this end, we evaluated the robustness of disease prioritization by computing the Spearman correlation between disease rankings obtained using the full set of Vitamin D targets and those derived from random subsamples. As expected, the correlation decreases with smaller target sets (Figure S5). Nonetheless, a Spearman correlation coefficient larger than 0.8 indicates that the rankings remain stable even when up to 20% of the targets are missing, highlighting the robustness of our results to moderate levels of target incompleteness. Furthermore, this analysis demonstrates NetMedPy's ability to support large-scale perturbation studies efficiently.

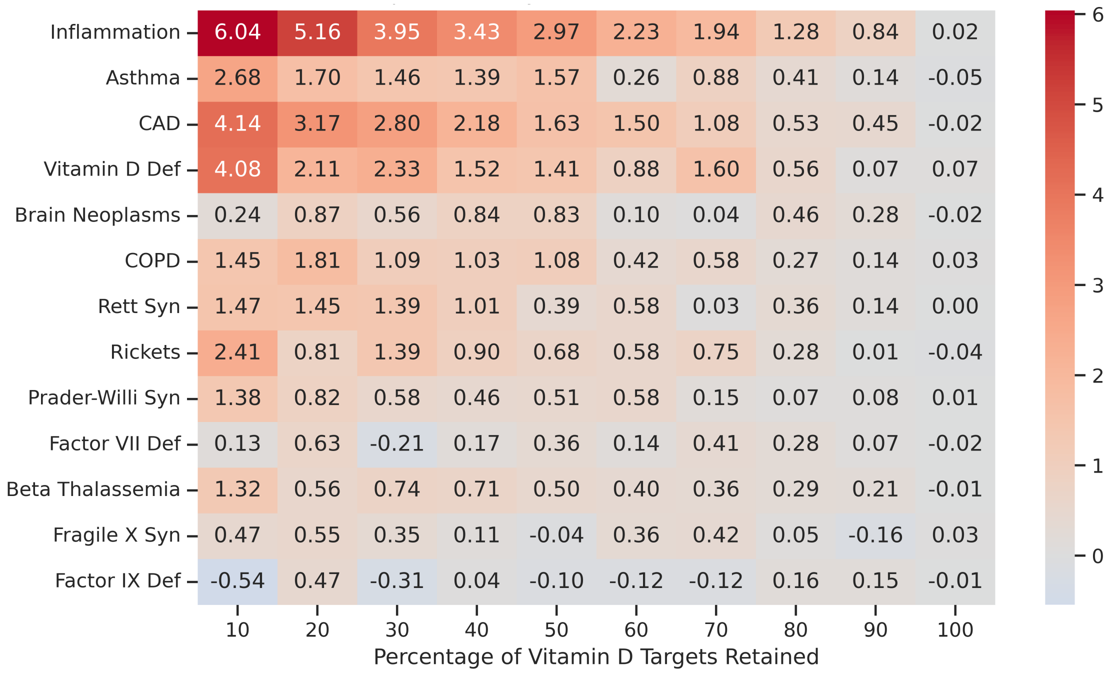

Figure S4: **Proximity deviations under Vitamin D target reduction.** In the heatmap, each row represents a disease and each column a sampling level, indicating the percentage of Vitamin D targets retained. The color intensity encodes $\Delta P_i(p)$ — the change in proximity relative to the full target set. Darker colors indicate stronger deviations.

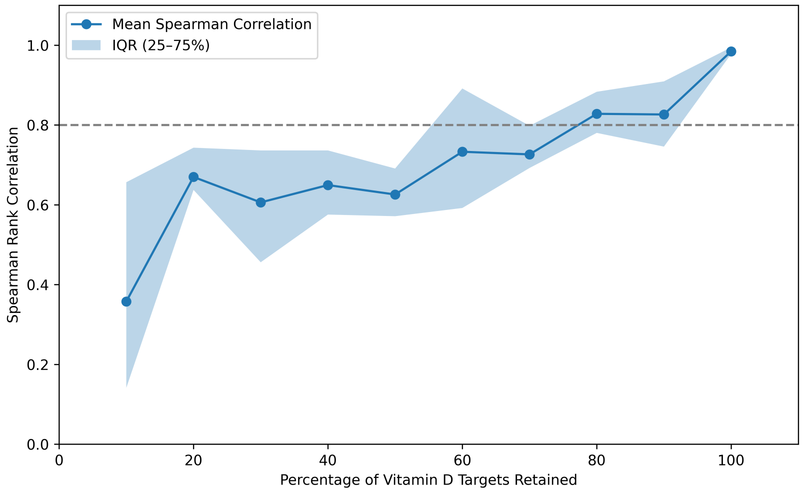

Figure S5: **Stability of disease ranking under random target reduction.** Spearman correlation between disease rankings computed with full Vitamin D target set vs. partial target subsets. High correlation values ($\rho > 0.8$) suggest that disease prioritization is largely robust to target incompleteness.

SVI. Supplementary Figures

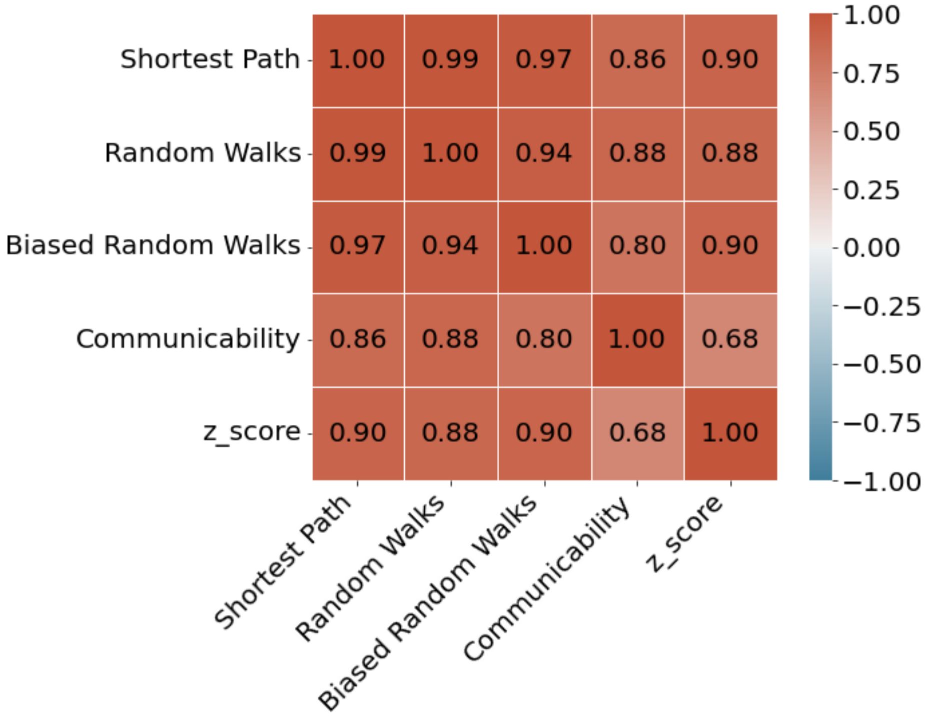

Figure S6: Agreement between the different metrics for the Vitamin D's AMSPL analysis. For the Vitamin D case study, we calculate the Pearson correlation among the AMSPL-equivalent raw values calculated with different metrics, including an additional comparison with the degree-preserving proximity Z-score calculated for the shortest path metric (Figure 1B). The correlation matrix demonstrates strong agreement between the metrics, indicating robustness in the results across different distance notions.

References

[1] A.-L. Barabási, N. Gulbahce, and J. Loscalzo. Network medicine: a network-based approach to human disease. *Nat Rev Genet.*, 12:56–68, 2011.

[2] L. Cowen, T. Ideker, B. J. Raphael, and R. Sharan. Network propagation: a universal amplifier of genetic associations. *Nature Reviews Genetics*, 18(9):551–562, 2017. doi: 10.1038/nrg.2017.38.

[3] S. Erten, G. Bebek, R. M. Ewing, and M. Koyutürk. DA DA: Degree-Aware Algorithms for Network-Based Disease Gene Prioritization. *BioData Min.*, 4(1):19, 2011. doi: 10.1186/1756-0381-4-19.

[4] E. Estrada and N. Hatano. Communicability in complex networks. *Phys Rev E.*, 77(3): 036111, 2008.

[5] E. Estrada, N. Hatano, and M. Benzi. The physics of communicability in complex networks. *Physics reports*, 514(3):89–119, 2012. doi: 10.1016/j.physrep.2012.01.006.

[6] E. Guney, J. Menche, M. Vidal, et al. Network-based in silico drug efficacy screening. *Nat Commun.*, 7:10331, 2016.

[7] E. L. Huttlin, R. J. Bruckner, J. Navarrete-Perea, J. R. Cannon, K. Baltier, F. Gebreab, M. P. Gygi, A. Thornock, G. Zarraga, S. Tam, et al. Dual proteome-scale networks reveal cell-specific remodeling of the human interactome. *Cell*, 184(11):3022–3040, 2021. doi: 10.1016/j.cell.2021.04.011.

[8] D.-H. Le. Random walk with restart: A powerful network propagation algorithm in bioinformatics field. In *2017 4th NAFOSTED Conference on Information and Computer Science*, pages 242–247. IEEE, 2017. doi: 10.1109/NAFOSTED.2017.8108071.

[9] K. Luck, D.-K. Kim, L. Lambourne, K. Spirohn, B. E. Begg, W. Bian, R. Brignall, T. Cafarelli, F. J. Campos-Laborie, B. Charloteaux, et al. A reference map of the human binary protein interactome. *Nature*, 580(7803):402–408, 2020. doi: 10.1038/s41586-020-2188-x.

[10] B. A. Maron, R.-S. Wang, S. Shevtsov, S. G. Drakos, E. Arons, O. Wever-Pinzon, G. S. Huggins, A. O. Samokhin, W. M. Oldham, Y. Aguib, et al. Individualized interactomes for network-based precision medicine in hypertrophic cardiomyopathy with implications for other clinical pathophenotypes. *Nature communications*, 12(1):873, 2021. doi: 10.1038/s41467-021-21146-y.

[11] N. Masuda, M. A. Porter, and R. Lambiotte. Random walks and diffusion on networks. *Phys Rep.*, 716-717:1–58, 2017. doi: 10.1016/j.physrep.2017.07.007.

[12] J. Menche, A. Sharma, M. Kitsak, et al. Uncovering disease-disease relationships through the incomplete human interactome. *Science*, 347(6224):1257601, 2015.

[13] J. J. Patten, P. T. Keiser, D. Morselli-Gysi, et al. Identification of potent inhibitors of SARS-CoV-2 infection by combined pharmacological evaluation and cellular network prioritization. *iScience*, 25(9), 2022.

[14] A. Piras, S. Chenghao, M. Sebek, et al. Cpiextract: A software package to collect and harmonize small molecule and protein interactions. *bioRxiv*, page 2024.07.03.601957, 7 2024. doi: 10.1101/2024.07.03.601957.

[15] A. Sharma, J. Menche, C. C. Huang, T. Ort, X. Zhou, M. Kitsak, N. Sahni, D. Thibault, L. Voung, F. Guo, et al. A disease module in the interactome explains disease heterogeneity, drug response and captures novel pathways and genes in asthma. *Human molecular genetics*, 24(11):3005–3020, 2015. doi: 10.1093/hmg/ddv001.
